# Rutin bioconjugates as potential nutraceutical prodrugs: An *in vitro* and *in ovo* toxicological screening

**DOI:** 10.3389/fphar.2022.1000608

**Published:** 2022-09-23

**Authors:** Cristina Adriana Dehelean, Dorina Coricovac, Iulia Pinzaru, Iasmina Marcovici, Ioana Gabriela Macasoi, Alexandra Semenescu, Geza Lazar, Simona Cinta Pinzaru, Isidora Radulov, Ersilia Alexa, Octavian Cretu

**Affiliations:** ^1^ Faculty of Pharmacy, “Victor Babes” University of Medicine and Pharmacy Timisoara, Timisoara, Romania; ^2^ Research Center for Pharmaco-Toxicological Evaluations, “Victor Babes” University of Medicine and Pharmacy Timisoara, Timisoara, Romania; ^3^ Faculty of Food Engineering, Banat's University of Agricultural Sciences and Veterinary Medicine “King Michael I of România”, Timişoara, Romania; ^4^ ”Ioan Ursu” Institute of the Faculty of Physics, Babes-Bolyai University, Cluj-Napoca, Romania; ^5^ Faculty of Agriculture, Banat's University of Agricultural Sciences and Veterinary Medicine “King Michael I of România”, Timişoara, Romania; ^6^ Faculty of Medicine, “Victor Babes” University of Medicine and Pharmacy Timisoara, Timisoara, Romania

**Keywords:** rutin oleate, rutin linoleate, bioavailability, toxicological profile, healthy cells, reconstructed human epidermis tissue, chick chorioallantoic membranes

## Abstract

Rutin (RUT) is considered one the most attractive flavonoids from a therapeutic perspective due to its multispectral pharmacological activities including antiradical, anti-inflammatory, antiproliferative, and antimetastatic among others. Still, this compound presents a low bioavailability what narrows its clinical applications. To overcome this inconvenience, the current paper was focused on the synthesis, characterization, and toxicological assessment of two RUT bioconjugates obtained by enzymatic esterification with oleic acid (OA) and linoleic acid (LA)—rutin oleate (RUT-O) and rutin linoleate (RUT-L), as flavonoid precursors with improved physicochemical and biological properties. Following the enzymatic synthesis in the presence of Novozyme® 435, the two bioconjugates were obtained, their formation being confirmed by RAMAN and FT-IR spectroscopy. The *in vitro* and *in ovo* toxicological assessment of RUT bioconjugates (1–100 µM) was performed using 2D consecrated cell lines (cardiomyoblasts - H9c2(2-1), hepatocytes—HepaRG, and keratinocytes—HaCaT), 3D reconstructed human epidermis tissue (EpiDerm™), and chick chorioallantoic membranes, respectively. The results obtained were test compound, concentration—and cell-type dependent, as follows: RUT-O reduced the viability of H9c2(2-1), HepaRG, and HaCaT cells at 100 µM (to 77.53%, 83.17%, and 78.32%, respectively), and induced cell rounding and floating, as well as apoptotic-like features in the nuclei of all cell lines, whereas RUT-L exerted no signs of cytotoxicity in all cell lines in terms of cell viability, morphology, and nuclear integrity. Both RUT esters impaired the migration of HepaRG cells (at 25 µM) and lack irritative potential (at 100 µM) *in vitro* (tissue viability >50%) and *in ovo* (irritation scores of 0.70 for RUT-O, and 0.49 for RUT-L, respectively). Computational predictions revealed an increased lipophilicity, and reduced solubility, drug-likeness and drug score of RUT-O and RUT-L compared to their parent compounds—RUT, OA, and LA. In conclusion, we report a favorable toxicological profile for RUT-L, while RUT-O is dosage-limited since at high concentrations were noticed cytotoxic effects.

## Introduction

Flavonoids stand as one of the largest groups of plant secondary metabolites (Corradini et al., 2011), being abundantly present in various plant-based foods such as fruits, vegetables, and herbs, as well as beverages including tea, wine, and juices (Ponte et al., 2021; Dehelean et al., 2021). Flavonoids fall into the category of benzo-γ-pyrone structured polyphenols and are subclassified as flavanols, flavonols, anthocyanins, isoflavones, flavanones or flavones depending on their chemical structure, unsaturation degree, and oxidation of carbon ring (Kumar and Pandey, 2013; Thilakarathna and Rupasinghe, 2013; Ullah et al., 2020). *In planta*, flavonoids serve as protectors against both biotic and abiotic hazards such as ultraviolet (UV) radiation and pathogens, also furnishing odor, flavor, or color to some species (Ponte et al., 2021). Additionally, the majority of flavonoids are widely embraced as therapeutic agents (Ullah et al., 2020), owing to their drug-like nature showing antioxidant, antiviral, antibacterial, antidiabetic, anti-inflammatory, anticancer, neuro-, hepato-, and cardio-protective properties (Abou Baker, 2022; Ullah et al., 2020; Kumar and Pandey, 2013).

Although flavonoids have displayed numerous bioactivities in different *in vitro* systems, the main drawback hindering their development as drug candidates remains the lack of *in vivo* validation (Thilakarathna and Rupasinghe, 2013; Moine et al., 2018). Most polyphenolic structures, including flavonoids, possess inconvenient bioavailability due to their physicochemical properties. Hydrophilic and highly polar compounds suffer from a restricted cell penetration, while those possessing lipophilic properties and a scarce water solubility, encounter absorption difficulties and/or high systemic metabolism following oral administration (Moine et al., 2018). The biological fate of flavonoids upon *in vivo* administration has been extensively researched (Crozier et al., 2010), several causes leading to unproper pharmacokinetics being listed, such as the interaction of flavonoids with the intestinal microflora, their first-pass metabolic conversion involving phase II enzymes which catalyze the production of highly hydrophilic conjugates (e.g., glucuronides, sulfates), and the involvement of efflux transporters fastening the removal of the resulting phase II conjugates (Hu et al., 2017; Thilakarathna and Rupasinghe, 2013).

Therefore, questions have been raised lately regarding the possible solutions for counteracting these biopharmaceutical drawbacks. A possible approach to increase bioavailability is by developing flavonoid precursors or prodrugs capable of withstanding transit through epithelia while releasing the parent compound once in circulation (Biasutto et al., 2007). Prodrugs are derivatives of pharmacologically active agents designed to overcome drug development challenges that limit formulation options or result in unacceptable pharmacokinetic performance, or poor targeting (Rautio et al., 2018). Prodrugs have evolved from being randomly discovered to being intentionally designed (Rautio et al., 2018), leading to the emergence of a significant fraction of marketed therapeutic formulations based on prodrugs (Walther et al., 2017). Within a prodrug, the pharmacological activity is temporary masked and recovered within the human body upon the prodrug bioconversion, a process that is mediated by enzymatic or chemical reactions (Rautio et al., 2018; Walther et al., 2017). The basic flavonoid skeleton contains numerous hydroxyl groups forming the main target for chemical or enzymatic derivatization using a more lipophilic substituent. The concept of flavonoid lipophilization expanded in the last few years, since by introducing lipophilic molecules to the original flavonoid structure not only refines the physicochemical properties but also improves the pharmacological profile of flavonoids (Danihelová et al., 2012; Chen and Yu, 2017). The most common route to obtain flavonoids’ derivatives with a greater lipophilicity is enzymatic esterification with fatty acids (Mecenas et al., 2018; de Araújo et al., 2017). Fatty acids (FAs) are biologically active molecules widely studied due to their effects on human health and disease (Baum et al., 2012). Besides playing energetic and structural roles within the human body, FAs have become a focus of recommendations for cardiac health (Iuchi et al., 2019; Baum et al., 2012). In particular, unsaturated fatty acids (UFAs) classified as monounsaturated fatty acids (MUFAs) or polyunsaturated fatty acids (PUFAs) based on the number of double bonds present within their chemical structure, are considered nutritionally valuable (Lee and Park, 2014). Oleic acid (OA) is the most abundant MUFA in human nutrition, while linoleic acid (LA) is the major dietary PUFA (Baum et al., 2012; Choque et al., 2014). OA exerts a cardioprotective function, its supplementation being correlated with a lower risk of heart diseases (Karacor, 2015). LA was described as an essential fatty acid (Choque et al., 2014; Serini et al., 2011), being associated with a beneficial effect on the cardiac function, as well as reduced levels of plasma low-density lipoprotein cholesterol and liver fat, and improved hepatic metabolic status (Marangoni et al., 2020). Despite the benefits, UFAs are highly unstable compounds with an increased aptitude for oxidative degradation (Kerrihard et al., 2015).

Rutin (RUT), also referred to as rutoside, sophorin, vitamin P, or quercetin-3-O-rutinoside, is a hydrophobic flavonol-type phytochemical, consisting of quercetin and rutinose (Patel and Patel, 2019; Pinzaru et al., 2021; Rashidinejad et al., 2022), and is one of the most attractive flavonoids from a pharmaceutical perspective owing to its multispectral pharmacological activities such as antiradical, anti-inflammatory, antiproliferative, and antimetastatic among others (Satari et al., 2021). Besides being usually recommended for the treatment of various conditions such as varicosities, and haemorrhoids (Yong et al., 2020), or skin disorders (e.g., skin aging, dermatitis, psoriasis) (Pinzaru et al., 2021), RUT is also included in various multi-vitamin preparations (Koval’skii et al., 2014). However, similar to its flavonoid counterparts, RUT suffers from a low bioavailability substantially limiting its pharmaceutical applications (Rashidinejad et al., 2022). The reduced bioavailability of RUT is determined by several intrinsic factors as: its poor water solubility, low stability, scant affinity for cell membranes and limited capacity for membranes penetration, characteristics that are essential for its *in vivo* biological effects. Moreover, RUT’s hydrophilic character narrows its use for topical applications (Gullon et al., 2017).

In this context, the lipophilic derivatization of RUT using unsaturated fatty acids might result in a double benefit, such as an enhancement of the bioavailability of the flavonoid component upon administration while conferring protection to the fatty acid constituent against undesirable oxidation.

In the light of the above considerations, the present study aims to offer a physicochemical, an *in vitro* and an *in ovo* toxicological evaluation of two unsaturated fatty acid-derived rutin bioconjugates, namely rutin oleate (RUT-O) and rutin linoleate (RUT-L), as potential nutraceutics for the pharmaceutical industry with improved biological properties in the prevention of cardiovascular, hepatic and skin disorders.

## Materials and methods

### Reagents and cell culture

Rutin, oleic acid (OA), linoleic acid (LA), Novozyme 435 (Immobilized *Candida antarctica* lipase), acetone, chloroform, methanol, molecular sieves, sodium dodecyl sulphate (SDS), trypsin-EDTA solution, phosphate saline buffer (PBS), dimethyl sulfoxide (DMSO), fetal calf serum (FCS), penicillin/streptomycin, insulin from bovine pancreas, hydrocortisone 21-hemisuccinate sodium salt, and MTT [3-(4,5-dimethylthiazol2-yl)-2,5-diphenyltetrazolium bromide] reagent were purchased from Sigma Aldrich, Merck KgaA (Darmstadt, Germany). Dulbecco’s Modified Eagle Medium (DMEM; ATCC® 30–2002™) was acquired from ATCC (American Type Culture Collection, Lomianki, Poland), while William’s Medium E was provided by Gibco (12551032, Waltham, MA, United States).

### Cell culture

Cardiomyoblasts—H9c2(2-1) (CRL-1446™) were acquired as a frozen vial from ATCC. Keratinocytes—HaCaT were purchased from CLS Cell Lines Service GmbH (300493, Eppelheim, Germany), and hepatocytes—HepaRG (HPRGC10) from ThermoFisher Scientific (Waltham, MA, United States). The cells were cultured in their specific media—H9c2(2-1) and HaCaT in DMEM; HepaRG in William’s E Medium supplemented with 4 μg/ml insulin from bovine pancreas and 50 µM hydrocortisone 21-hemisuccinate sodium salt. Both culture media contained 10% FCS and 1% penicillin (100 U/ml)-streptomycin (100 μg/ml) mixture. The cells were incubated at 37°C and 5% CO_2_ during the experiments. All three cell lines expressed normal growth features under standard culture conditions.

### Rutin biocatalytic acylation

The rutin/fatty acid bioconjugates were obtained by enzymatic esterification as described by de Araujo et al. with slight modifications (de Araújo et al., 2017). Shortly, the biosynthesis was conducted in an orbital shaker following several steps: 1) addition of rutin and the unsaturated fatty acids to the reaction medium (acetone, previously dried on 4 Å molecular sieves, at room temperature for 5 days) for solubilization (under magnetic stirring, at 50°C, for 10–12 h); 2) utilization of the molar mass ratio flavonoid: acyl donor 1-5; 3) initiation of the enzymatic esterification with the introduction into the reaction mass of the immobilized enzyme followed by stirring in an orbital shaker with well-predetermined reaction conditions (temperature 50°C, 250 rpm, reaction time 120 h); 4) keeping a water content in the reaction mass below 0.3% with the help of molecular sieves; 5) monitoring the formation of reaction products by thin layer chromatography (TLC) by using the eluent system chloroform/methanol/water (80/20/0.3, v/v/v); 6) purification of esters by chromatographic column separation, and the analysis of the collected fractions by TLC; the fractions that indicated only the presence of bioconjugates were subjected to dry evaporation and recrystallization.

### Vibrational FT-Raman and FT-IR spectra

Fourier Transform Raman (FT-Raman) spectra were acquired using an Equinox 55 Bruker spectrometer with an integrated FRA 106S Raman module. A Nd:YAG laser operating at 1,064 nm was used for the excitation with an output power of 350 mW. A Ge detector operating at liquid nitrogen temperature was used for detection. For data acquisition, 350 scans were co-added. The spectral resolution was 2 cm^−1^.

Fourier Transform—Infrared (FT-IR) spectra were acquired using the KBr (potassium bromide) pellet technique in the 400–4,000 cm^−1^ spectral range with a spectral resolution of 4 cm^−1^.

### Cellular viability evaluation

To evaluate the *in vitro* effect of rutin esters (REs) on cells’ viability, the MTT technique was applied following a 24 h treatment of H9c2(2-1), HepaRG, and HaCaT cells with RUT-O and RUT-L (1, 10, 25, 50, and 100 µM). The parent compounds (PCs)—RUT, OA, and LA were also tested. DMSO was used as solvent for all test compounds (RUT, OA, LA, RUT-O, and RUT-L). The impact of DMSO at tested concentrations (1, 10, 25, 50, and 100 µM) was also assessed *in vitro*. At the end of the treatment, fresh media (100 µl) and the MTT reagent (10 µl) were added in each well, and the plates were incubated for 3 h at 37°C. The final steps were as follows: addition of the solubilization solution (100 µl/well), incubation of the plates at room temperature for 30 min, protected from light, and absorbance measurement at two wavelengths (570 and 630 nm) using Cytation 5 (BioTek Instruments Inc., Winooski, VT, United States). The results were expressed as percentage (%) of viable cells normalized to control cells (unstimulated cells).

### Cellular morphology and confluence assessment

To verify the impact of PCs (RUT, OA and LA) and REs (RUT-O and RUT-L) on the morphology and confluence of H9c2(2-1), HaCaT, and HepaRG cells, a microscopic examination was performed. The cells were observed under bright field illumination and photographed at the end of the 24 h treatment period using Cytation 1 (BioTek Instruments Inc., Winooski, VT, United States). The photographs were processed using the Gen5™ Microplate Data Collection and Analysis Software (BioTek Instruments Inc., Winooski, VT, United States).

### Nuclear morphology evaluation

The potential toxicity of 100 μM RUT, OA, LA, RUT-O, and RUT-L at nuclear level was tested by applying the Hoechst 33342 staining assay protocol according to the manufacturer’s (Thermo Fisher Scientific, Inc., Waltham, MA, United States) recommendations. In brief, H9c2(2-1), HepaRG, and HaCaT cells were seeded in 12-well plates (10^5^ cells/1.5 ml/well) and treated with the test compounds for 24 h. After the stimulation period, the media was removed, and the staining solution diluted 1:2000 in PBS was added (500 µl/well). The plates were incubated for 10 min at room temperature, protected from light. Finally, the staining solution was washed with PBS and the pictures were taken using Cytation 1 (BioTek Instruments Inc., Winooski, VT, United States) and analyzed by the means of Gen5™ Microplate Data Collection and Analysis Software (BioTek Instruments Inc., Winooski, VT, United States). Staurosporine (STP) 5 µM was selected as positive control for apoptosis.

### Wound healing assay

The influence of RUT-O and RUT-L on the migration of cells was assessed by performing the wound healing (scratch) assay. In brief, the cells (10^5^ cells/ml/well) were cultured in 24-well plates (Corning Costar), and an automatic scratch was made in each well by using the AutoScratch™ Wound Making Tool provided by BioTek® Instruments Inc. (Winooski, VT, United States). The cells were further treated with the flavonoid esters at the concentration of 25 µM for 24 h, and representative images of the wound area were taken at 0 and 24 h using Cytation 1 (BioTek® Instruments Inc., Winooski, VT, United States). The wound widths were measured using Gen5 ™ Microplate Data Collection and Analysis Software (BioTek® Instruments Inc., Winooski, VT, United States). The effects on cell migration were quantified by calculating the wound healing rates (%) using a formula presented in our previous studies (Farcas et al., 2020).

### *In vitro* skin irritation potential

The reconstructed human epidermal model—EpiDerm™ (EPI-212, MatTek Corporation protocol, 2021, Bratislava, Slovakia) (Lot no. 36155) is a multilayered differentiated human epidermis model made of normal human-derived epidermal keratinocytes, validated as test method for the OECD 439 (*In vitro* skin irritation: reconstructed human epidermis test methods) (OECD, 2021). The EpiDerm™ tissues (prepared as inserts) that served as model for the skin irritation test together with the specific culture media (assay medium—EPI-100-NMM), Dulbecco’s phosphate buffered saline (DPBS) (negative control), sodium dodecyl sulphate (SDS) 5% and the MTT kit were provided by the manufacturer (MatTek Corporation, Bratislava, Slovakia).

The *in vitro* skin irritation test (EPI-212-SIT) was performed according to the manufacturer’s recommendations and to the data from the literature [MatTek Corporation protocol—*In vitro* EpiDerm Skin Irritation Test (EPI-200-SIT); Kandarova et al., 2018]. In brief, the following steps were carried out: 1) day 0—upon receipt, the inserts with tissues were cleaned of agarose and visually inspected, transferred into 6-well plates filled with 0.9 ml/well of assay medium for 1 h at 37°C and following the incubation period (1 h) the culture medium was replaced and the inserts were incubated overnight for 24 h; 2) day 1—the assay medium was renewed and the test samples were added on the top of the inserts (30 µl/insert of test solutions—RUT, OA, LA, RUT-O and RUT-L) and incubated for 1 h; after the incubation, the inserts were rinsed with sterile DPBS and incubated for 24 h; 3) day 2—after the 24 h incubation, the inserts were moved into fresh culture medium (0.9 ml/well) for another 18 h; 4) day 3—the inserts were transferred into a 24-well plate filled with MTT solution (1 mg/ml—0.3 ml/well) and incubated for 3 h at 37°C; following MTT incubation, each insert was immersed into 2 ml of isopropanol (the extractant solution), the plate was sealed and gently shaked for 2 h at room temperature; after the extraction period the absorbance values were read at 570 nm using the Cytation 5 (BioTek Instruments Inc., Winooski, VT, United States). The same protocol was performed for the positive (5% SDS in saline) and negative (sterile DPBS) controls. The tissue viability (%) was calculated according to the formulas specified in the literature (Pinzaru et al., 2021).

### *In ovo* irritant potential

To perform a toxicological screening and to verify the irritant potential of the RUT-O and RUT-L were used chicken eggs (*Gallus domesticus*). Specifically, the eggs were prepared as follows: 1) after being washed and disinfected with 70% ethanol (v/v), the eggs were placed in an incubator in a horizontal posture; 2) on the fourth day of incubation, a small hole was made through which a volume of approximately 7–8 ml of albumen was extracted in order to facilitate the detachment of the chorioallantoic membrane from the inner shell of the egg; 3) on the fifth day of incubation, a window was cut in the upper part of the egg to allow the visualization of the blood vessels, which was then covered with adhesive tape and the eggs were placed in the incubator until the day of the beginning of the experiment.

Using the Hen’s Egg Test—Chorioallantoic Membrane (HET-CAM) assay, the potential *in ovo* toxicity of the samples was evaluated. This experiment was conducted according to the protocol described above, involving the following steps: 1) on the 10th day of incubation, at the vascular plexus of the chorioallantoic membrane, RUT-O and RUT-L, at a concentration of 100 µM along with a positive control (sodium dodecyl sulphate, 1%) and a negative control - distilled water, were applied; 2) a volume of 600 μl of each sample was added in order to ensure the complete coverage of the chorioallantoic membrane; 3) the following changes in the blood vessels were noted through a 5 minutes monitorization: hemorrhage (H), lysis (L) and coagulation (C); 4) for each sample, the time of the vascular changes was noted, then irritation was calculated according to the formula (Batista-Duharte et al., 2016; Kis et al., 2022).

$$IS=5\times \frac{301-H}{\mathrm{300}}+7\times \frac{301-L}{\mathrm{300}}+9\times \frac{301-C}{\mathrm{300}}$$

In order to observe any vascular effects, a stereomicroscope (Discovery 8 Stereomicroscope Zeiss, Göttingen, and Germany) was used, and images were taken with a Zeiss Axio CAM 105 color camera before and 5 min after the application of the samples. The same experimental protocol was applied for the parent compounds—RUT, OA, and LA.

### Computational prediction of drug-likeness properties and potential toxicity

In addition to the tests described above, in the current work, the open-source program OSIRIS Property Explorer was selected to predict the potential drug-like nature and toxicity of RUT, OA, LA, RUT-O, and RUT-L. The program provides an estimation of the compounds’ toxic events (i.e., mutagenic, tumorigenic, irritant, and reproductive), as well as other relevant properties such as the molecular weight (MW), cLogP, solubility, and drug-likeness. Furthermore, by combining these aforementioned parameters, an overall drug score is generated which shows the compound’s potential of becoming a possible drug candidate (Ayati et al., 2012). The canonical SMILES (simplified molecular-input line-entry system) used in this computational assessment were obtained from https://pubchem.ncbi.nlm.nih.gov/.

### Statistical analysis

All data are expressed as means ± SD (standard deviation), the differences being compared by applying the one-way ANOVA analysis followed by Dunett’s multiple comparisons post-test. The used software was GraphPad Prism version 9.2.0 for Windows (GraphPad Software, San Diego, CA, United States, www.graphpad.com). The statistically significant differences between data are marked with * (*p < 0.05; **p < 0.01; ***p < 0.001; ****p < 0.0001).

## Results

### Vibrational spectroscopy of bioconjugates

**FIGURE 1:**
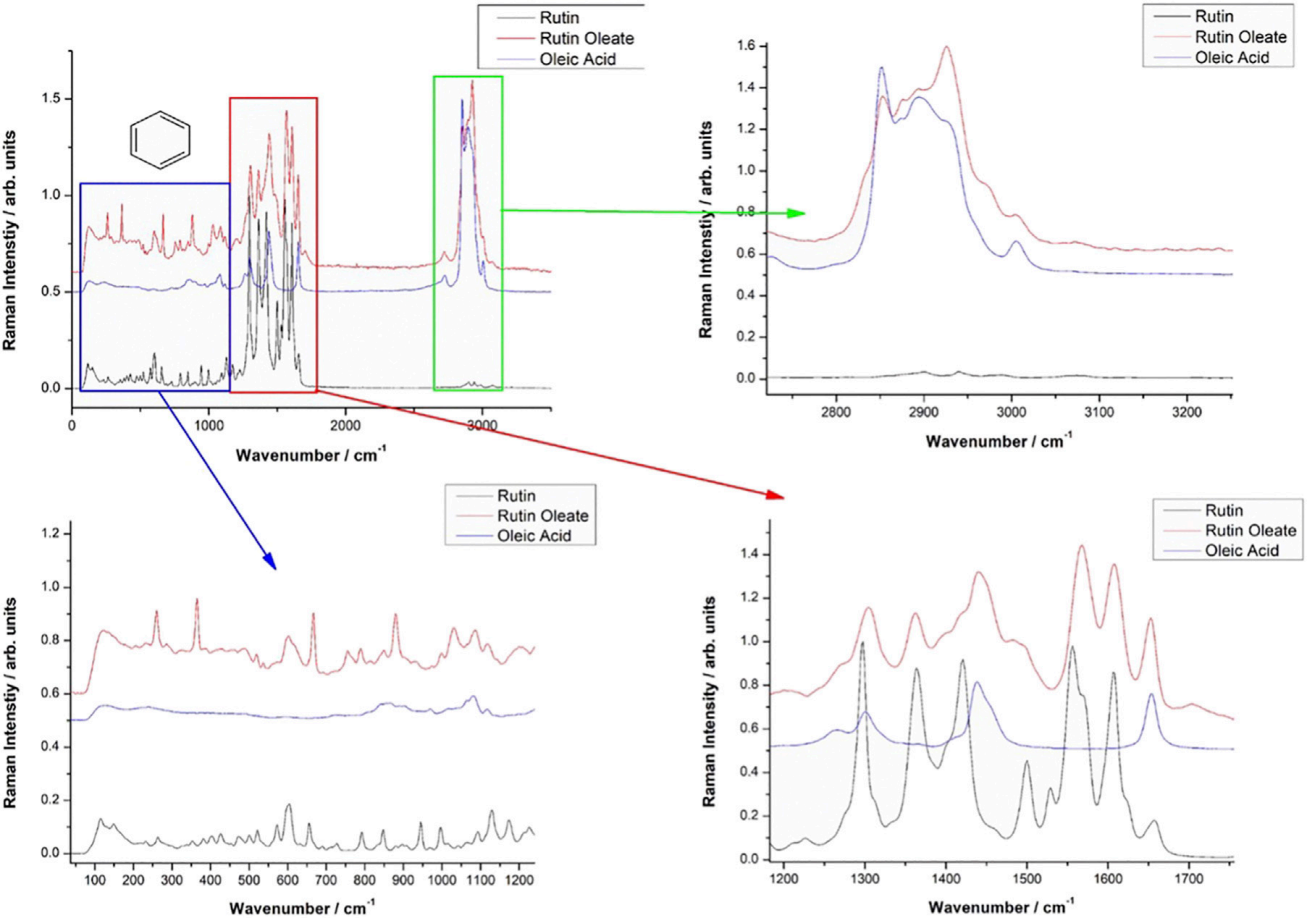
Comparative Raman spectra of Rutin (RUT), Oleic acid (OA) and Rutin oleate (RUT-O).

**FIGURE 2:**
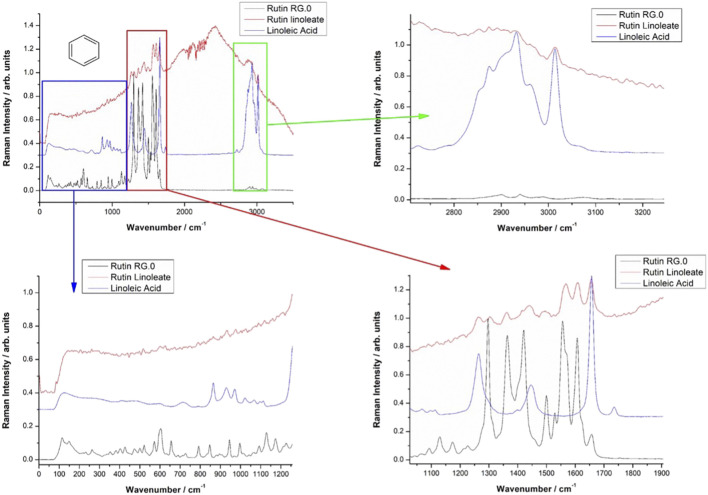
Comparative Raman spectra of Rutin (RUT), Linoleic acid (LA) and Rutin linoleate (RUT-L).

Vibrational FT-Raman spectra of RUT, OA and RUT-O are showed in Figure 1, while the corresponding linoleate bioconjugate is comparatively showed in Figure 2. When adding OA and LA to RUT, it is expected that the Raman spectra of the bioconjugate compounds (RUT-O and RUT-L) to exhibit the strong bands of OA and LA, respectively, as well as the characteristic skeletal modes of RUT, with a small shift characteristic to the corresponding ester. Vibrational Raman spectra of RUT, OA, and LA have been compared to the spectra of RUT-O and RUT-L esters, respectively. The spectra of the parent compounds are in agreement with the previous data (Krysa et al., 2022). As previously showed, OA possesses the main Raman bands at 602(w), 725(w), 845 (m, sh), 856(m), 866(m), 890(m), 903(m), 971(m), 1,023(m, sh), 1,035(m, sh), 1,065(m), 1,080(m), 1,118(m), 1,265(m), 1,301(s), 1,416(m,sh), 1,440(vs.), and 1,655(s) (De Gelder et al., 2007). The spectra of oleate/linoleate esters are indeed dominated by the characteristic bands of the parent compounds, with several small changes, mainly characteristic to the vibrational modes change on passing from RUT to its corresponding esters. The details of the specific spectral ranges are comparatively given in Figures 1, 2. For RUT-O, in the fingerprint spectral range (1,000–1,800 cm^−1^) there are 6 intense bands, each one could be attributed to vibrations present either in RUT or OA. The position of the bands is slightly shifted in RUT-O compared to RUT and OA. In the 50–800 cm^−1^ region the OA does not present any intense bands, the peaks visible in the spectrum of RUT-O can be attributed to the complex vibration modes of the skeletal rings from the RUT molecular structure. The position of the peaks is slightly shifted. Moreover, in the high wavenumber region ∼3,000 cm^−1^, the spectral signature of the O-H stretching mode can be seen with peaks observable both in RUT and OA. Although it seems that OA bands in this spectral region are more intense than the RUT bands, we can observe the contribution of both, in the RUT-O spectra, as a shift of the peak positions as well as a change in relative intensities (Figure 1). The same trends are visible in the case of RUT-L as expected, due to the similar nature of the OA and LA (Figure 2).

The FT-IR data support the Raman spectroscopy findings (not showed here). Briefly, the FT-IR spectra of OA are dominated by strong bands at 1,711 cm^−1^, characteristic to the C=O bond in carboxylic acid, a complex band shape from multiple contributions in the 1,416–1,466 cm^−1^, three medium band at 1,216, 1,248, and 1,284 cm^−1^, and another two medium bands at 939 and 721 cm^−1^. In the high wavenumber range, two sharp IR bands at 2,855 and 2,926 cm^−1^ from CH_2,3_ stretching mode with a shoulder at 2,953 cm^−1^ are characteristic for the fatty acid. An additional weak band at 3,006 cm^−1^ is also present, usually assigned to the C-H stretching from aromatic rings. The distinct FT-IR feature of LA compared to that of OA is represented by the strong band at 1,734 cm^−1^ and a relatively stronger band at 1,187 cm^−1^.

The FT-IR strongest bands of RUT are observed at 1,002, 1,014, 1,043, 1,058, 1,202, 1,239, 1,297, 1,365, 1,459, 1,503, 1,600, and 1,658 cm^−1^ and in the high wavenumber range, a complex-shaped band in the 2,850–1,944 cm^−1^ region and three bands at 3,178, 3,327, and 3,422 cm^−1^ are observed.

The FT-IR data of RUT-O showed the strongest band at 1,711 cm^−1^ with two shoulders at 1,695 and 1,737 cm^−1^, while the feature of RUT-L showed prominent bands at 1,714 and 1,733 cm^−1^, along with a series of medium bands attributable to the parent compounds. These prominent features highlight the ester vibrational data, slightly shifted from those prominent bands of the respective fatty acid.

FT-IR spectra of the RUT-O are dominated by the strong band at 1,713 cm^−1^ with a shoulder at 1,737 cm^−1^ indicating the presence of the C=O bond in ester. The C-O band in ester is observed as a new, medium band at 1,270 cm^−1^ which is absent in the FT-IR spectrum of RUT.

### RUT-O and RUT-L influence cells’ viability dependent on cell type and concentration

**FIGURE 3:**
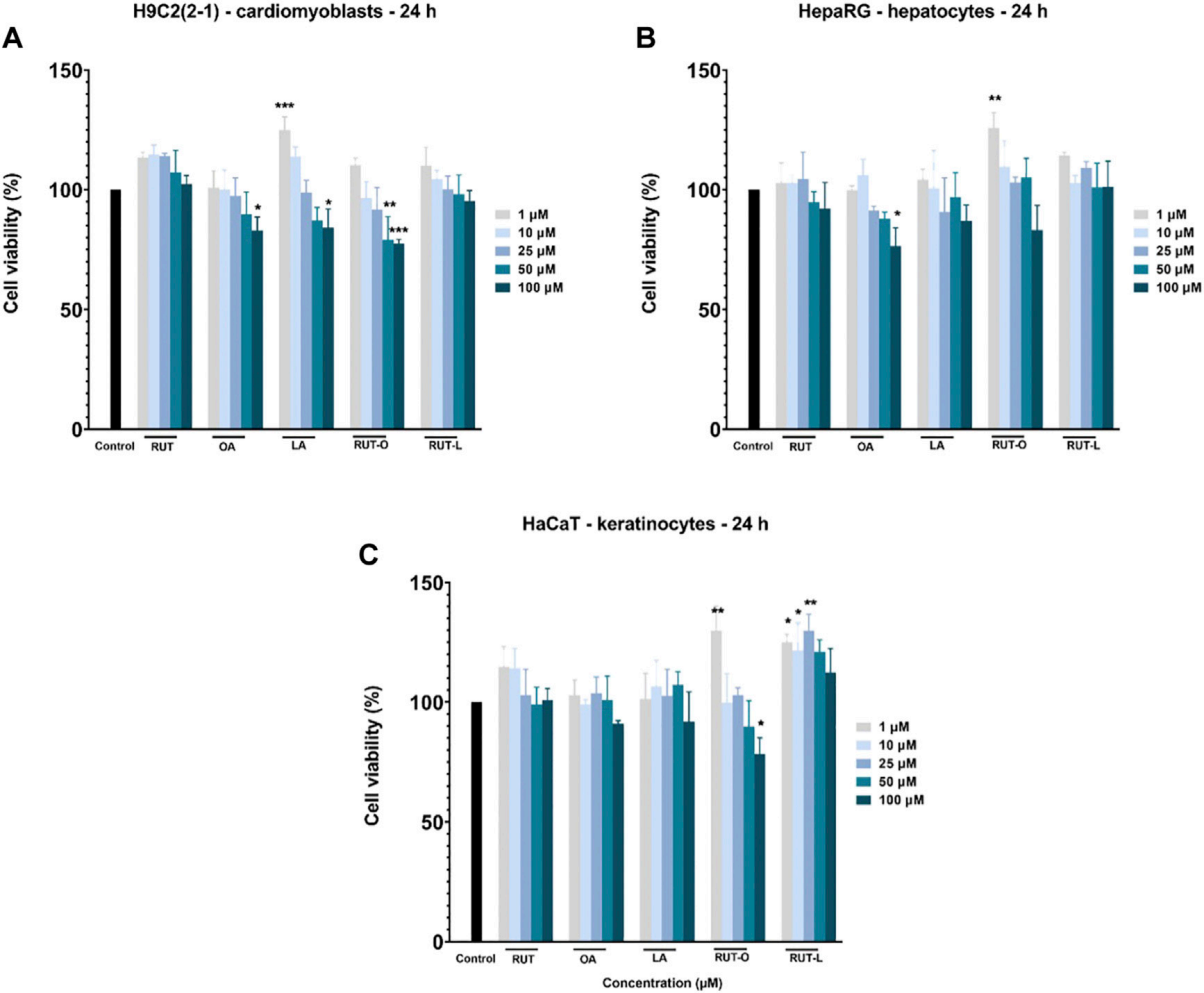
*In vitro* cell viability evaluation of the parent compounds (PCs) and rutin esters (REs) in healthy **(A)** cardiomyoblasts—H9c2(2-1), **(B)** hepatocytes - HepaRG, and **(C)** keratinocytes - HaCaT cells after 24 h of treatment by performing the MTT assay. Five concentrations were selected for this assay—1, 10, 25, 50, and 100 µM. Data are presented as viability percentages (%) normalized to control (untreated cells) and are expressed as mean values ±SD of three independent experiments performed in triplicate. The statistical differences between control and the treated group were verified by applying the one-way ANOVA analysis followed by Dunnett’s multiple comparisons post-test. The statistically significant differences between data are marked with * (*p < 0.05; **p < 0.01; ***p < 0.001).

The impact of the synthesized REs (RUT-O and RUT-L) on the viability of healthy cardiomyoblasts—H9c2(2-1), hepatocytes - HepaRG, and keratinocytes—HaCaT was examined by applying the MTT assay. The obtained results were compared to control (untreated cells). A wide concentration range (from 1 to 100 µM) was selected for a 24 h treatment. Additionally, the cytotoxic potential of the PCs (RUT, OA, and LA) has been evaluated. According to the obtained viability results (Figure 3), the response of cells to test compounds treatment was dependent on the concentration tested and the type of cell. The most sensitive cells to the highest concentrations (50 and 100 µM) of all test compounds except for RUT-L, were the hepatocytes—HepaRG, the lowest cell viability percentages being calculated for OA (87.86% and 76.43%, respectively) (Figure 3B). LA also induced a decrease of HepaRG viability at the highest concentration (100 μM—86.89%), whereas the ester RUT-L had no cytotoxic impact on this type of cells (100 μM—101.25%). In the case of RUT-O, the percentage of viable HepaRG cells was reduced only at 100 µM (83.17%), indicating that the ester is less cytotoxic as its parent compounds—RUT and OA.

The cardiomyoblasts—H9c2(2-1) were affected by the highest concentrations (50 and 100 µM) of OA (89.80 and 82.90%), LA (87.18 and 84.21%) and RUT-O (79.03 and 77.53%), whereas RUT and RUT-L did not decrease cells viability. Moreover, the lowest concentrations of RUT, LA, and RUT-L (1 and 10 µM) had a stimulatory effect (Figure 3A). The less sensitive cells to test compounds treatment were the keratinocytes—HaCaT, being induced a statistically significant decreased viability percentage only by RUT-O—100 µM (78.32%). The other compounds even at the highest concentration have not reduced the percentage of viability under 90%. An interesting finding was that RUT-L exerted a stimulatory effect at all concentrations (Figure 3C).

### RUT-O and RUT-L Induced Slight Changes in Cells’ Morphology

**FIGURE 4:**
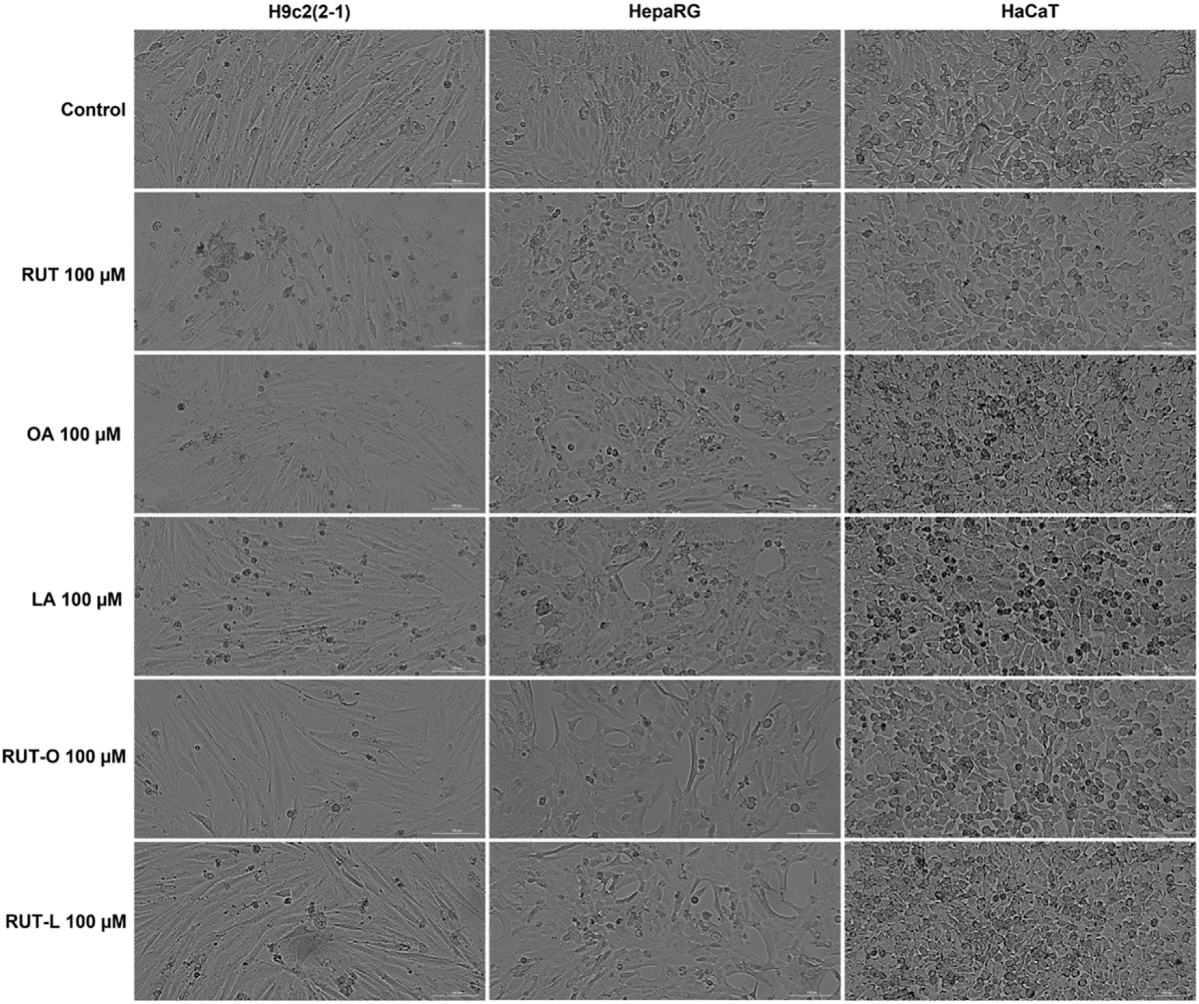
Microscopical appearance of cellular morphology and confluence of H9c2(2-1), HepaRG, and HaCaT cells following a 24 h treatment with the parent compounds (RUT, OA, LA), and rutin esters (RUT-O, RUT-L) at 100 µM. The scale bars represent 200 µm.

To visualize the morphology and confluence changes in H9c2(2-1), HepaRG, and HaCaT cells following the 24 h treatment with RUT, OA, LA, RUT-O, and RUT-L at the concentration of 100 μM, a bright field microscopical examination was conducted (Figure 4). The H9c2(2–1) cells displayed a myoblast-like morphology and reached the confluence at 48 h after plating, forming a uniform monolayer. HepaRG cells presented a hepatocyte-like morphology with polygonal shapes and needed 48 h for reaching a complete confluent monolayer. In the case of HaCaT—immortalized keratinocytes, the confluency was achieved after 24 h. The 24 h treatment with the test compounds (RUT, OA, LA, RUT-O, and RUT-L—100 µM) had no negative impact on cells’ adherence or confluency as compared to control cells (untreated cells), still there were noticed several round cells floating in the culture medium in the groups treated with OA, LA, and RUT-O (Figure 4), data that are consistent with the cell viability results.

### RUT-O induced apoptotic-like signs in all cells

**FIGURE 5:**
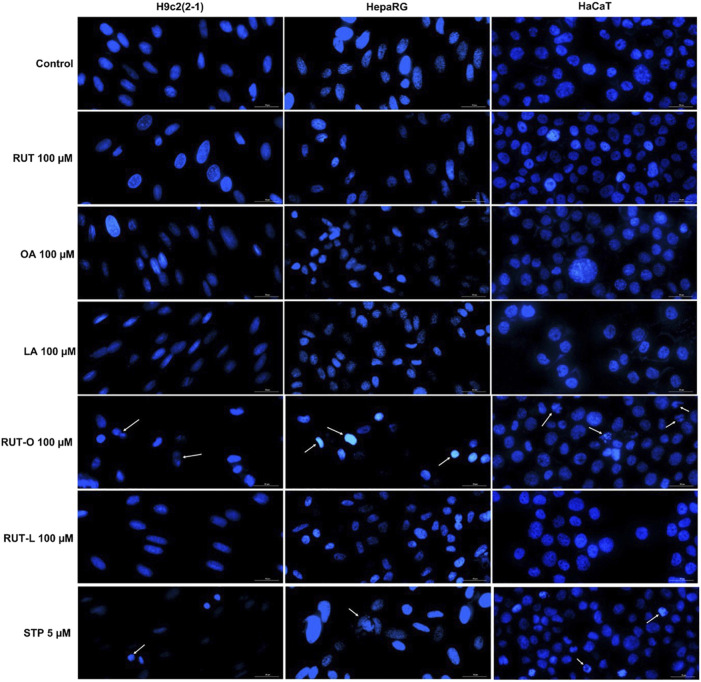
Nuclear staining of H9c2(2-1), HepaRG, and HaCaT cells following a 24 h treatment with the parent compounds (RUT, OA, LA) and rutin esters (RUT-O, RUT-L) at 100 µM. Staurosporine (STP) 5 µM was used as positive control for apoptosis. White arrows indicate nuclei presenting an apoptotic-like aspect. The scale bars represent 30 µm.

Taking into consideration the decreased cells’ viability percentages calculated at the highest concentration—100 μM, it was decided to verify the potential morphological changes occurring at nuclear level following the 24 h treatment with the PCs and REs by performing the Hoechst 33342 staining assay. Staurosporine (STP) 5 µM was chosen as apoptosis inductor. Compared to untreated cells (control) and STP, several apoptotic-like features in the cells treated with RUT-O 100 µM were captured via fluorescence microscopy (Figure 5). RUT-O generated chromatin condensation in HepaRG cells, as well as nuclear fragmentation and formation of apoptotic bodies in HaCaT cells. No significant changes in the nuclear aspect of H9c2(2-1), HepaRG, and HaCaT cells were recorded following their treatment with RUT-L 100 μM, being consistent with our previous results. PCs (RUT, OA, and LA) at 100 µM also induced no significant nuclear alterations in all cells.

### RUT-O and RUT-L impair cell migration in a cell-type manner

**FIGURE 6:**
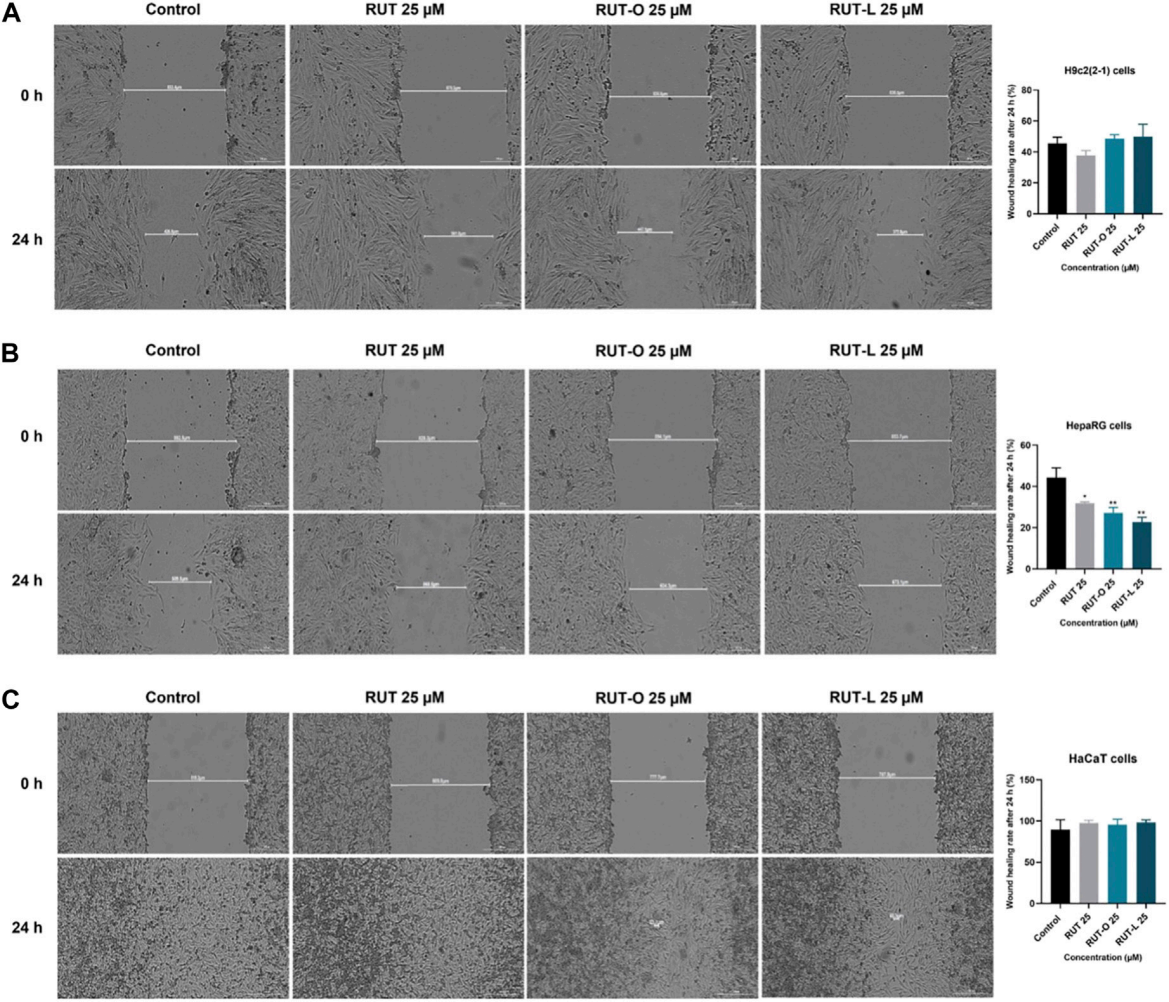
Representative images illustrating the impact of RUT, RUT-O and RUT-L 25 µM on wound regeneration in **(A)** H9c2(2-1) **(B)** HepaRG and **(C)** HaCaT cells following a 24 h treatment and graphic representations of the calculated wound healing rates. The scale bars represent 300 µm. The data are expressed as mean values ±SD of three independent experiments performed in triplicate. The statistical differences between the control and the treated group were quantified by applying the one-way ANOVA analysis followed by the Dunett’s multiple comparisons post-test (*p < 0.05; **p < 0.01).

To verify whether RUT, RUT-O, and RUT-L impair cell migration, a median and non-toxic concentration (25 µM) was selected for implementing the wound healing assay. According to our results, the impact of the REs on cell migration is cell type dependent (Figure 6). Thus, RUT-O and RUT-L did not interfere with the migration of H9c2(2-1) cells, while RUT slightly inhibited their migratory ability (wound healing rate of 37.73% compared to control—45.71%). On the other hand, both REs significantly inhibited the migration of HepaRG cells (wound healing rates of 27.18% and 22.77% for RUT-O and RUT-L 25 µM compared to control—44.31% and RUT 25 μM–31.83%). In HaCaT cells, no impact on wound regeneration has been detected following the 24 h treatment with RUT and both REs (wound healing rates were similar to control).

### RUT-O and RUT-L classify as non-irritant to reconstructed human epidermis tissues

**FIGURE 7:**
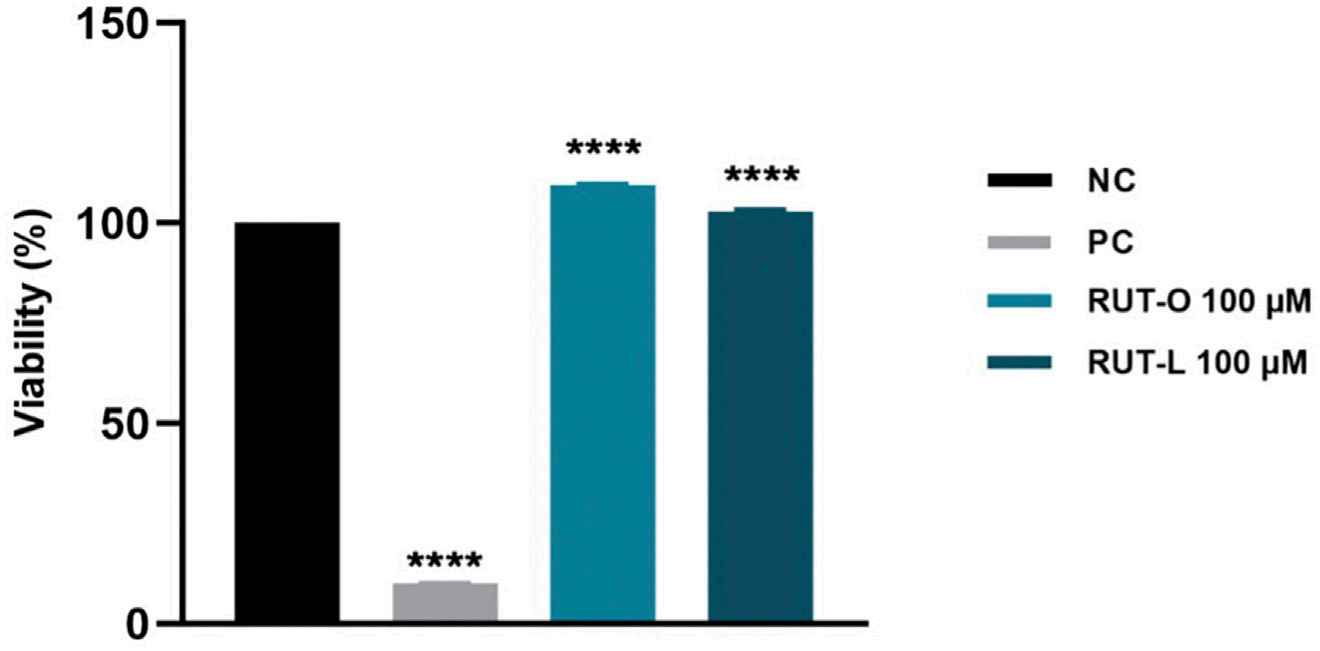
Viability percentage of EpiDerm skin model inserts (EPI-212-SIT) following treatment with RUT-O and RUT-L—100 µM. One-way analysis of variance (ANOVA) followed by the Dunett’s post-test was employed to determine the statistical differences between sample-treated inserts and negative control-treated inserts (*p* < 0.0001 indicated by ****). Negative control (NC) is represented by DPBS, and positive control (PC) is represented by SDS 5%.

The *in vitro* toxicological screening of REs was complemented by a skin irritation test performed with reconstructed human epidermis 3D tissues—EpiDerm™ (EPI-212). According to OECD Test Guideline 439, a sample is considered irritant if the viability of the sample-treated insert is below 50% after employing the skin irritation test. In our case, RUT-O and RUT-L (100 µM) showed a slight proliferative effect, the viabilities being maintained over 100% compared to negative control (DPBS-treated inserts). Similar results were obtained for RUT, OA, and LA, tested at the same concentration as the esters (data not shown). On this basis, it can be concluded that since the viability of the treated-insert exceeded 50%, both REs and PCs can be classified as non-irritant (Figure 7). The positive control used was SDS 5% solution that induced a decrease of tissues viability percentage up to 10%.

### RUT-O and RUT-L displayed no *in ovo* irritant potential

**FIGURE 8:**
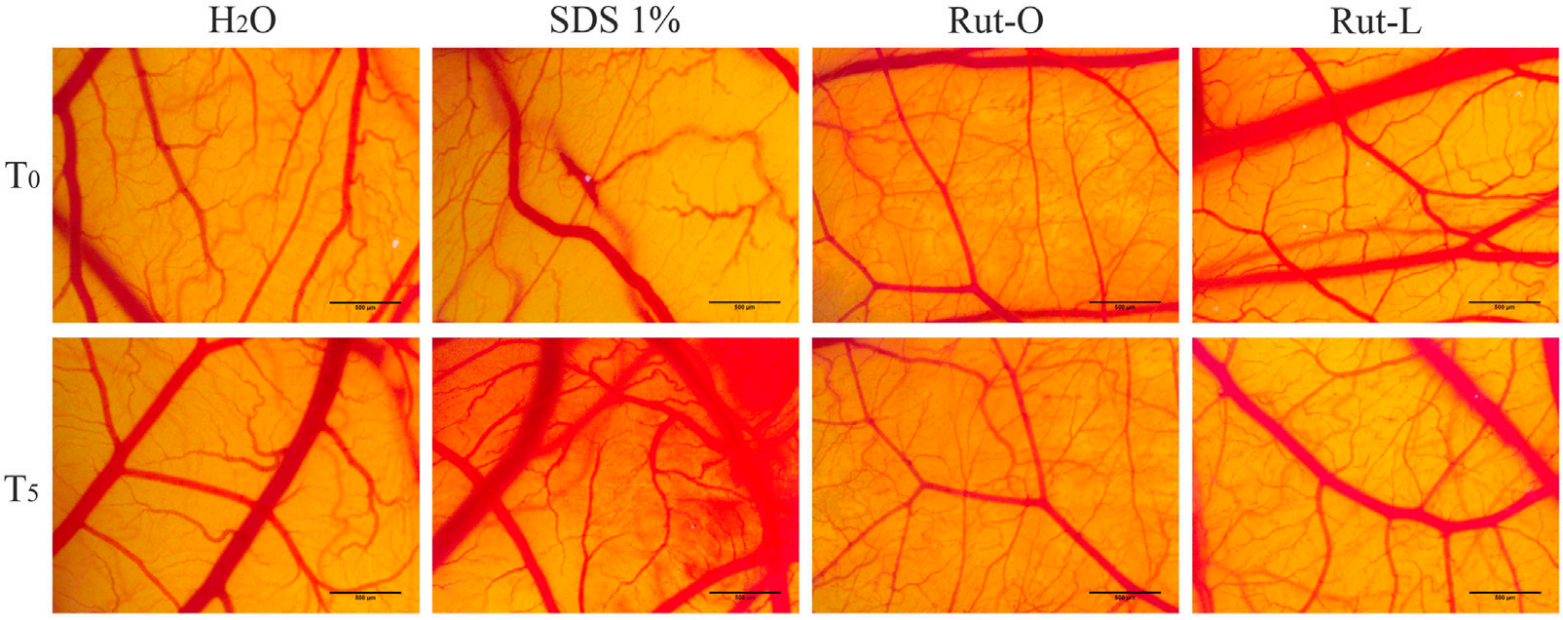
*In ovo* analysis of the irritant potential of RUT-O and RUT-L 100 μM by applying the HET-CAM method. Stereomicroscopic images of CAMs inoculated with negative control—H_2_O, positive control—SDS 1% and test compounds—RUT-O and RUT-L 100 μM. The scale bars represent 500 µm.

In addition to the *in vitro* skin irritation test, the *in ovo* HET-CAM method was employed. RUT-O and RUT-L were applied to the chorioallantoic membranes, and the changes observed for 5 minutes were compared to the negative and positive controls, respectively. Table 1 summarizes the irritation scores obtained for both the tested compounds and the two controls. The highest irritation score was obtained for the SDS 1% solution, 19.78. Conversely, the negative control, represented by water, scored the lowest irritation score, 0.10. Regarding the irritation scores obtained for the two compounds tested, these were 0.70 for RUT-O, and 0.49 for RUT-L, respectively. Additionally, Figure 8 presents images of chorioallantoic membranes before and at 5 min after sample application. SDS 1% exhibited the most obvious signs of vascular irritation, manifested by the development of extensive areas of vascular hemorrhage, as well as lysis and coagulation of blood vessels. Accordingly, no significant irritant effects have been observed in the blood vessels following the administration of the RUT-O and RUT-L. No signs of vascular irritation characterized by hemorrhage, lysis, and coagulation, were detected for PCs—RUT, OA, and LA (data not shown).

**TABLE 1:** Irritation score (IS) values for positive control (SDS 1%), negative control (distilled water), RUT-O, and RUT-L. tH = time of hemorrage; tL = time of lysis; tC = time of coagulation.

	H_2_O	SDS 1%	RUT-O	RUT-L
IS	0.10	19.78	0.70	0.49
tH	300	22 s	300	300
tL	300	20 s	291	295
tC	299	15 s	286	290

### Drug-likeness properties—Computational predictions

The properties of RUT, OA, LA, RUT-O, and RUT-L predicted via OSIRIS Property Explorer are presented in Table 2. According to the results, REs possess higher MW and cLogP values compared to RUT, while their solubility is lower than that of their parent compound. REs were found to possess a negative drug-likeness and a lower drug score compared to RUT. By verifying all these parameters, among the two tested REs, RUT-L exerted slightly better drug-like properties than RUT-O. OA and LA expressed similar properties in terms of MW, lipophilicity, solubility, drug-likeness, and drug score. The analyzed molecules showed no risk for mutagenic, tumorigenic, irritant, or reproductive toxicity (Roseblade et al., 2017).

**TABLE 2:** Computational prediction of the properties exerted by RUT, OA, LA, RUT-O, and RUT-L by applying OSIRIS Property Explorer.

Compound	MW	cLogP	Solubility	Drug-likeness	Drug score	Toxic potential			
						Mutagenic	Tumorigenic	Irritant	Reproductive
RUT	610	−1.26	−2.4	3.31	0.57	No risk	No risk	No risk	No risk
OA	282	6.72	−4.55	−28.9	0.22				
LA	278	6.21	−4.09	−20.7	0.25				
RUT-O	874	6.25	−6.9	−28.6	0.09				
RUT-L	858	5.54	−6.4	−20.4	0.1				

## Discussions

Rutin is considered one of the most interesting nutraceutical molecules on account of its diversity of pharmacological properties involving different molecular mechanisms (antioxidative, anti-inflammatory, anticancer, cardioprotective, hepatoprotective, and antidiabetic, etc.) and of its multiple health benefits. Nonetheless, RUT’s clinical application is restricted by some limitations as: a low solubility in aqueous solutions and an impaired pharmacokinetic profile, parameters that affect its bioavailability (Semwal et al., 2021). In considering this, the present study addressed the following goals: 1) to increase RUT’s bioavailability by improving its lipophilicity via enzymatic esterification with unsaturated fatty acids (OA and LA) and obtention of RUT/fatty acids esters that will be used as potential flavonoid precursors (prodrugs) for preventive and/or curative applications; 2) to offer a complete physicochemical characterization of the synthetized RUT esters; 3) to perform an *in vitro* and *in ovo* toxicological assessment using established 2D cell cultures (cardiomyoblasts, hepatocytes, keratinocytes), 3D reconstructed human epidermal tissues, and the chick chorioallantoic membrane model for the experimental validation of the esters in terms of toxicity as compared to parent compounds and, lastly 4) to assess the drug-likeness properties of the esters by computational predictions study as background for further extensive studies.

To improve RUT’s hydrophobic nature, stability, and bioavailability, in this study were synthetized two esters/bioconjugates of rutin—RUT-O and RUT-L by enzymatic esterification, more precisely by acylation with unsaturated fatty acids. The enzymatic acylation also known as biosynthesis was selected based on the following considerations: 1) functional hydroxyl groups which serve as primary targets for derivatization are abundantly found in the structure of RUT; 2) the number and substitution of hydroxyl groups influence the bioavailability, biological activity, and metabolism of flavonoids (Dias et al., 2021; Kumar and Pandey, 2013); 3) endogenous esterases facilitating the metabolic regeneration of drugs are ubiquitously found within the human body (Walther et al., 2017) and 4) enzymatic acylation is regioselective and might enhance both its physicochemical features and biological effects (Gullon et al., 2017; De Oliveira et al., 2009; Viskupicova and Maliar 2017).

Compared to chemical synthesis, biosynthesis, due to the enzymes used as catalysts (biocatalysts), has several advantages, among which the following should be mentioned: it is both enantiomeric and regioselective, and can be applied to complex processes without the need for additional steps of protection or deprotection of the compounds; requires normal working temperatures; can take place in the pH range 2–12; no secondary reaction products are obtained, etc. (Wu et al., 2021; Bell et al., 2021).

To correct the drawbacks that characterize the flavonoids (low solubility and stability), a series of attempts were performed including enzymatic esterification of these molecules with fatty acids, in the presence of biocatalysts such as proteases and lipases, under various operating technologies. The major challenge in the case of flavonoid acylation is the achievement of improved stability and solubility without affecting the pharmacological action. Enzymatic acylation is an optimal method from this point of view, being regioselective, easy to apply and inexpensive. The most common biocatalysts are immobilized enzymes that have two major advantages, namely: 1) high selectivity, stability, reactivity ensuring accessibility to the catalytic site of the enzyme and 2) the possibility of reuse, being very easy to recover (SreeHarsha et al., 2019). Immobilized *Candida antarctica* lipase (especially Novozym® 435) is considered to be the most effective enzyme in the case of flavonoids acylation, as multiple synthesis were performed so far, like: rutin with vinyl acetate (Mecenas et al., 2018), naringin, hesperidin, rutin with monocarboxylic acids (de Araújo et al., 2017), naringin and neohesperidin with fatty acids (Sun et al., 2017), quercetin-3-O-Glucoside with fatty acids (Warnakulasuriya and Ziaullah Rupasinghe, 2014), naringin with fatty acids (Lee et al., 2022).

In this study, the RUT concentration was established considering its poor solubility in organic solvents and the performed syntheses showed that an excess of fatty acid is needed for the good progress of the biosynthesis, which is in accordance with the existing data in the literature (Viskupicova, 2010; de Araújo et al., 2017). A flavonoid/fatty acid molar ratio of 1/5 was used, while higher concentrations of RUT in the reaction medium did not increase the final yield, and productivity can be significantly reduced due to low solubility (de Araújo et al., 2017). FT-IR spectra reproduce the characteristic bands of flavonoids, unsaturated fatty acids and their bioconjugates. The analysis of purified esters by FT-IR and Raman spectroscopy confirmed the formation of the products, usually a single reaction product, in the form of a monoester (acylation that took place at the hydroxyl in the C-4‴ position), due to the regioselectivity of the enzyme (De Oliveira et al., 2009; Viskupicova, 2010).

Besides the pharmacological spectra of RUT that recommends it for clinical use, another important feature is represented by its lack of toxicity verified in rats, rabbits, and guinea pigs, being considered safe for consumption along with other flavonoids (Semwal et al., 2021; Thakur et al., 2019). Furthermore, supplementation of unsaturated fatty acids, such as OA and LA, is generally considered advantageous for human health, especially due to their cardiovascular benefits (Karacor, 2015; Marangoni et al., 2020; Lopez-Huertas, 2010). However, to the best of our knowledge, RUT’s oleic and linoleic derivatives lack a rigorous toxicological verification so far. Considering a potential therapeutic application of REs as prodrugs, a series of experimental evaluations were performed in the present study to portrait their toxicological profile, reflecting the following toxicity endpoints: *in vitro* cellular viability impairment, cellular morphological alterations, nuclear integrity disruptions, inhibition of cell migration capacity, skin irritant potential, as well as *in ovo* irritative potential.

The *in vitro* toxicological screening of RUT-O and RUT-L was performed on three different healthy cell lines (cardiomyoblasts—H9c2(2-1), hepatocytes—HepaRG, and keratinocytes—HaCaT) by comparison with the parent compounds—RUT, OA, and LA. These cell lines were selected on the following basis: 1) are established *in vitro* models used for the investigation of toxicity at cardiovascular, hepatic and cutaneous levels, respectively; 2) represent the target organs and tissues of RUT’s biological effects (including cardio-protective, hepato-protective, and skin-oriented beneficial properties) (Siti et al., 2020; Domitrović et al., 2012; Choi et al., 2016), and 3) will be further used as reliable models to assess the pharmacological effects of REs.

Our cell viability results after a 24 h treatment showed a dose -, cell-type and test compound-dependent cytotoxic effect, as follows: 1) RUT-O decreased significantly the percentage of viable cells only at the highest concentrations tested (50 and 100 µM) in the following order: H9c2(2-1)> HaCaT > HepaRG cells (Figure 3); 2) by contrast, RUT-L had no toxic impact in all tested cell lines, moreover the low concentrations induced a proliferative effect (Figure 3); 3) RUT slightly reduced HepaRG cells viability only at 100 μM, whereas H2c9(2-1) and HaCaT cells were not affected by its effect; 4) OA significantly decreased cells’ viability at the highest concentrations tested (50 and 100 µM), as follows: HepaRG > H9c2(2-1)> HaCaT cells, and 5) in the case of LA, the susceptibility of cells at 50 and 100 µM was HaCaT > H9c2(2-1)>HepaRG cells (Figure 3). These results could be explained by the different features of each cell-type tested and of each test compound. H9c2(2-1) cells are morphologically close to immature embryonic cardiomyocytes, possess a good proliferation *in vitro*, and are applied as reliable tools in cardiotoxicity investigations (Rednic et al., 2022; Witek et al., 2016; Zordoky and EL-Kadi, 2007). These cells present a spindle shape, sugar residues on the cell surface, parallel arrangement, and the capacity to differentiate into multinuclear myotubes. In addition, H9c2(2-1) cells possess voltage dependent Ca^2+^ channels, a particularity of cardiac cells (Kobuszewska et al., 2017). Furthermore, H9c2 cells have been widely employed as a model system for *in vitro* investigations regarding the protective mechanisms associated with flavonoids (Daubney et al., 2015). Similarly, the human immortalized hepatocytes—HepaRG are widely employed as models for various *in vitro* assays, including toxicity evaluations (Klein et al., 2014; Rednic et al., 2022). Advantageously, the HepaRG cell line maintains the major hepatic-like functions (as drug transporters and enzymes involved in xenobiotics metabolism), have the ability to differentiate into hepatocyte-like and biliary-like cells (Tascher et al., 2019; Young and Young, 2019) and represent a powerful alternative to primary human hepatocytes with higher availability and reproducibility (Rednic et al., 2022; Hu et al., 2019; Blidisel et al., 2021). HaCaT cells are spontaneous immortalized human keratinocytes with a long life in culture that express basal properties and could be differentiated under the effects of different inducers as Ca^2+^ and high cell density. Moreover, transplantation of these cells onto athymic mice led to the formation of a nearly normal epithelial structure (Colombo et al., 2017; Wilson, 2014). HaCaT cell line also proved its applicability as model in toxicity testing (Ridd et al., 2010).

To the best of our knowledge, there is no previous *in vitro* research screening the toxicological impact of RUT-O and RUT-L on healthy cell lines. However, other flavonoid/fatty acid derivatives were investigated. As regards the more elevated cytotoxic potential of RUT-O compared to RUT-L, a similar observation has been made by Kubiak-Tomaszewska et al. whose study revealed that oleic derivatives of 6-hydroxy-flavanone and 7-hydroxy-flavanone (0–100 µM) had an increased cytotoxic effect both in HaCaT keratinocytes and PC3 prostate cancer cells following a 72 h treatment compared to the linoleic conjugates and to the parent compounds (Kubiak-Tomaszewska et al., 2022). In other study, OA and LA esters of quercetin-3-O-glucoside were shown to possess no cytotoxicity to human primary hepatocytes, while exerting a cytoprotective effect by overcoming the H_2_O_2_-induced damage (Warnakulasuriya and Ziaullah, 2016). Similarly, Moine et al. observed that LA introduction to quercetin did not lead to an enhanced toxicity in retina pigment epithelial (ARPE-19) up to 160 μM, while conferring a protective activity to the obtained lipophenol (Moine et al., 2018). Quercetin-3-Oleate exerted a stimulatory effect on the proliferation of HaCaT cells following 72 h of treatment (Carullo et al., 2019). Mecenas et al. showed that RUT monoacetate and diacetate derivatives obtained by transesterification reactions catalyzed by Novozym 435 exerted a lower cytotoxicity in healthy cells (RAW 264.7 macrophages, and Vero kidney epithelial cells) after 24 h of treatment compared to RUT (Mecenas et al., 2018). The pronounced cytotoxic potential of RUT-O compared to RUT, could be explained by the increased lipophilicity of the ester, what could determine a more facile penetration of the compound through cellular membrane and a modulation of different signaling pathways, hypotheses that requires further studies.

According to our data, RUT showed a slight toxicity against HepaRG cells at the highest concentrations used (50 and 100 µM), whereas the H9c2(2-1) and HaCaT cells were not affected (Figure 3), what indicates a cell-type and concentration-dependent cytotoxic effect. These data are supported by other studies, as follows: 1) no significant impact was induced by RUT in H9c2(2-1) cells (up to 100 μM, after 24 h of treatment) or hepatocytes (up to 500 μg/ml, after 24 and 48 h of treatment) (Jeong et al., 2009; El-Maadawy et al., 2021); 2) a low cytotoxicity was described in RAW 264.7 macrophages and Vero kidney epithelial cells after 24 h treatment (Mecenas et al., 2018); 3) a significant reduction of RAW 264.7 cells’ viability starting with 100 µM (Choi et al., 2021); 4) an inhibitory effect on HaCaT cells’ growth (IC_50_ = 60 μg/ml) (Deepika et al., 2019); 5) a significant decrease in cell viability of Vero normal kidney cells at 100 and 250 µM after a 48 h treatment (Caparica et al., 2020), and 6) a reduced cell viability of human dermal fibroblasts (HDFs) starting with 50 µM after 24 h exposure (Choi et al., 2016). Moreover, in our previous study concentrations of RUT as high as 75 µM induced a proliferative effect in HaCaT cells (Pinzaru et al., 2021).

The cytotoxic effects of OA and LA at high concentrations seen in the present study, were also described by other authors in different healthy cells, as: 1) OA and LA induced an inhibitory effect on primary bovine satellite cells (BSCs) viability at 250 μM, while enhancing their proliferation at a lower concentration (100 µM) following a 24 h treatment (Belal et al., 2018); 2) LA induced a 10% decrease in the viability of trophoblast-like Swan71 cells compared to vehicle control following 24 h of treatment (Shrestha et al., 2019); 3) LA suppressed HUVEC endothelial cells growth only at concentrations higher than 300 µM (Lu et al., 2010); 4) OA induced cytotoxicity in HUVEC endothelial cells at doses above 200 µM (Bass et al., 2020); 5) no toxic effect in THLE-2 human hepatocytes was induced by OA at doses of 300 µM (Giulitti et al., 2021).

The cells’ viability studies were complemented with cellular morphology and nuclear staining assays to confirm the cytotoxic effect of RUT-O and RUT-L compared to control, RUT, OA, and LA. The microscopical investigation of the cells after test compounds treatment indicated the presence of round and floating cells only after RUT-O, OA, and LA at 100 μM, but no impact on cells’ adherent properties or confluence were noticed (Figure 4). Since apoptotic cells can be distinguished from both healthy and necrotic cells through their specific morphological features (e.g., cell rounding, membrane blebbing, nuclear fragmentation and condensation) (Doonan and Cotter, 2008), bright field and fluorescence microscopical analyzes were conducted. However, the Hoechst staining—a technique allowing cell death detection in the nucleus via fluorescence microscopy (Ziegler and Groscurth, 2004), has revealed several morphological hallmarks of apoptosis in the nuclei of H9c2(2-1), HepaRG, and HaCaT cells following their treatment with RUT-O 100 μM, namely chromatin condensation, nuclear dysmorphology and fragmentation into apoptotic bodies (Figure 5, white arrows). Compared to control (untreated cells), RUT-L 100 µM induced no changes in the morphology of H9c2(2-1), HepaRG, or HaCaT cells’ nuclei, data that is consistent with the viability results indicating its lack of *in vitro* toxicity towards healthy cell lines.

Cell migration is a key process involved in many physiological events including wound repair which is essential for the restoration of structurally damaged tissues (Pijuan et al., 2019; Grada et al., 2017). *In vitro* analysis of cellular motility is useful to quantify alterations of cell migratory capacity in response to chemical agents (Mouritzen and Jenssen, 2018). Thus, in an attempt to examine whether the synthesized flavonoid esters at a low and non-toxic concentration (25 µM) interfere with cell migration, a wound healing assay was performed. Compared to control and RUT 25 μM, RUT-derived esters potently inhibited the migration of HepaRG cells, while preserving the migratory ability of HaCaT cells, and slightly stimulating regeneration following wounding in H9c2(2-1) cells (Figure 6). The different response of the cells to test compounds could be explained by their different morphology, confluency, and doubling time, data that were also noticed in cell viability studies. An inhibitory effect on HepG2 cells’ migration (a cell line that presents similar features to HepaRG cells) was observed after treatment with quercetin (Abruzzese et al., 2021). Quercetin-3-oleate was found to stimulate HaCaT wound healing up to 51% at a low concentration (1 μM) (Carullo et al., 2019). A stimulatory effect on HaCaT cells’ migratory capacity was induced by LA (Manosalva et al., 2020).

Regardless of its skin-oriented properties (e.g., antioxidant, antiaging, sunscreen) (Ganeshpurkar and Saluja, 2017), RUT cannot be efficiently used in dermocosmetic formulations given to its inadequate solubility and skin penetration rate (Pyo et al., 2016; Cândido et al., 2018). Fortunately, FAs have been shown to interact with the *stratum corneum* (SC) lipids, acting as skin permeation enhancers. Unsaturated FAs have demonstrated an increased promotion of skin penetration when compared to saturated FAs with the same chain length (Ibrahim and Li, 2010). Thus, RUT’s bioconjugation with unsaturated FAs might enhance its skin penetration and therefore its topical pharmaceutical effect. However, the potential cutaneous toxic events of the resulted products should be considered, since skin toxicity assessment is an essential component of the overall analysis of chemical substances and pharmaceutical products (Schmidt et al., 2020). Our previous report suggested that RUT lacks irritant and phototoxic effects when individually administered or applied as proniosomal gel in 3D human reconstructed epidermis (Pinzaru et al., 2021). In a recent clinical study, it has been shown that RUT appliance as topical formulation lacks (photo)irritant, allergic and photosensitization potential (Tomazelli et al., 2018). FAs are important components of skin surface lipids and are currently used as raw materials in cosmetic products (Yang et al., 2020). The presence of FAs, including LA and OA, has been shown to confer beneficial properties (e.g., anti-inflammatory, anti-bacterial, moisturizing, and photoprotective against UV radiation) to skin products such as olive oil. However, in high concentrations, OA might interact with SC lipids, promoting skin barrier disruptions (Soimee et al., 2019).

Up to date, there is no characterization of RUT-O and RUT-L as regards their skin toxicity. Therefore, having these aspects into consideration, the next safety evaluation of REs resorted to a more complex *in vitro* system (3D reconstructed human epidermis - EpiDerm™) allowing the verification of their skin irritative potential. Skin irritation testing (SIT) reveals not only a product’s permeability through the SC, but also its ability to disrupt cellular integrity leading to local inflammatory responses (Pinzaru et al., 2021). Driven by the full ban of animal testing of cosmetics in 2013 by the EU Cosmetics Regulation (EC 1223/2009), novel trends in toxicity evaluations involving non-animal approaches are promoted (Cizauskaite and Bernatoniene, 2018). A such ethical alternative allowing a comprehensive cutaneous toxicity testing is represented by the *in vitro* reconstructed human skin models developed via tissue engineering techniques (Schmidt et al., 2020). The reconstituted human epidermis is a multidimensional model that closely resembles the normal human epidermis in terms of biochemical and physiological properties (Pinzaru et al., 2021) and has been validated for *in vitro* SIT (Pedrosa et al., 2017) able to trace cellular behavior following irritation, inflammation and cell death onset (Pinzaru et al., 2021). Based on our findings, both REs and PCs (at 100 µM) can be considered non-irritant, since the viability of the reconstructed epidermal tissues exceeded 50% following treatment (Figure 7).

In addition to the Epi-Derm™ testing, *in ovo* assessments were conducted to confirm the irritant potential of RUT-O and RUT-L, since data on the mucous membrane irritation effect represent an important tool in hazard identification of chemicals. The HET-CAM method has become widely embraced as a substituent of the *in vivo* Draize test, being extensively applied to evaluate the irritation potential of various substances (Lanzerstorfer et al., 2021). At the same time, it is a simple method of classifying substances based on the irritation score, as follows: non-irritating substances (IS = 0–0.9), irritating substances (IS = 1–8.9) and strongly irritating substances (IS = 9–21) (Budai et al., 2021). The compounds were tested at a concentration of 100 μM, the highest concentration previously tested in the cell viability test. On the basis of the results obtained after evaluating the two REs, it can be stated that they have low irritation values, between 0.49 and 0.70, thus qualifying them as non-irritating substances (Table 1; Figure 8). A previous study using chorioallantoic membranes have been carried out to evaluate the effect of OA and LA on angiogenesis and it was observed that these two compounds were involved in the initiation of blood vessel formation (Samson et al., 2020). As far as we know, the irritant potential of RUT on the chorioallantoic membrane - as well as those of its esters - have not been investigated.

Finally, a computational analysis was conducted to predict the toxicity risks and drug-likeness of each ester, as well as of their parent compounds RUT, OA, and LA (Table 2). As expected, the lipophilicity of RUT (cLogP) increased, while its solubility decreased following esterification. According to the Lipinski’s rule of 5 (Ro5) developed to set ‘drugability’ for novel molecular entities, poor absorption and permeation is more likely to occur for compounds possessing MW > 500, and cLogP >5 (Benet et al., 2016). In this situation, both REs fail in complying to these conditions. However, violations of the Ro5 have been found among several existing drugs (e.g., antibiotics, antifungals, cardiac glycosides) which are still orally bioavailable owing to the presence of structural groups acting as substrates for transporters (Kadam and Roy, 2007). By comparing the tested REs, the computational predictions indicated a higher lipophilicity (cLogP) and lower solubility for RUT-O than for RUT-L. Correlated to the viability and Hoechst 33342 results, it can be assumed that the increased hydrophobic degree results in enhanced cell membrane crossing and thus a slightly elevated toxicity of the OA-derivative towards healthy H9c2(2-1), HepaRG, and HaCaT cells in comparison to the LA-conjugate, highlighted via reduced cell viability and nuclear dysmorphology. Similar conclusions were expressed by other researchers observing a possible link between the lipophilic properties of flavonoid conjugates, their cellular uptake, and consequent cytotoxicity (Danihelová et al., 2013; Kubiak-Tomaszewska et al., 2022). Viskupicova et al. showed that the lipophilic derivatization of rutin using fatty acids (such as OA and LA, among others) effectively increases its hydrophobicity and solubility in fats (Viskupicova, 2010). In a recent review, the authors also summarized several studies revealing that lipophilic rutin derivatives possess improved bioavailability and penetration through cell membrane (Viskupicova and Maliar, 2017). RUT, OA, and LA showed higher drug-likeness and drug scores compared to REs, since any slight structural alternation might affect the activity of flavonoids (Kubiak-Tomaszewska et al., 2022), and MW increasing is often correlated with an improved lipophilicity but also with a loss in drug-like properties (Naylor et al., 2018), suggesting the application of REs not as drug candidates, but as drug precursors capable of releasing the original active flavonoid following absorption. All molecules (RUT, OA, LA, RUT-O, and RUT-L) were predicted as being free of mutagenic, tumorigenic, irritant, and reproductive toxicity.

## Conclusion

The current paper aimed at obtaining RUT bioconjugates with improved physicochemical properties as potential flavonoid prodrugs for preventive and/or curative applications and at investigating the biosafety and drug-like properties of the two RUT-derived esters by applying *in vitro*, *in ovo* and computational methods. The primary findings of the current research revealed a favorable toxicological profile of both REs as discovered via experimental approaches which indicate a lack of substantial toxicity *in vitro* against different cell types (cardiac, hepatic, and skin) and absence of *in vitro* and *in ovo* irritative activity. Linoleate derivatization of RUT generates a bioconjugate with increased *in vitro* safety even at high concentrations, which, although limited in terms of drug-likeness compared to its PCs, possesses potential usage as flavonoid prodrug or active agent in preserving cellular homeostasis, integrity, and healing. Comparatively, the usage of the oleate derivative is dosage-limited since at high concentration toxic events might occur. These data represent the background for future pharmacological evaluations to confirm their potential as nutraceutics in the prevention of cardiovascular, hepatic and skin disorders.

## Data Availability

The original contributions presented in the study are included in the article/Supplementary Material, further inquiries can be directed to the corresponding author.
